# Actinomycosis in Man

**Published:** 1886-07

**Authors:** 


### EDITORIAL.

### ACTINOMYCOSIS IN MAN.

An editorial in the *New York Medical Record*, of March 20th, 1886, on "A New Form of Lung Disease," states that almost all cases of actinomycotic phthisis so far observed, have been found in Germany, yet it is not improbable that the disease occurs in this country, since the researches of Dr. W. T. Belfield have shown the prevalence of the actinomyces in American cattle. "It is to be hoped that American physicians living in the West, where opportunities for observing this disease occur, will report any cases that come under their observation."

The editor of the *Record* has probably not seen a report of two cases of human actinomycosis, read at a meeting of the Chicago Medical Society, on December 15th, 1884. An account of the proceedings of that meeting is published in the March, 1885, issue of the CHICAGO MEDICAL JOURNAL AND EXAMINER, page 259.

The reporter, Dr. J. B. Murphy, of this city, seems to have been the first to demonstrate the presence of the actinomyces in the human subject, in this country, and nearly two years in advance of the suggestion made in the *Record*.

